# A Real-World Pharmacovigilance Study of FDA Adverse Event Reporting System Events for Avelumab

**DOI:** 10.7759/cureus.81285

**Published:** 2025-03-27

**Authors:** Lu He, Xinyue Zhang, Yanrong Li, Rongrong Li, Encun Hou

**Affiliations:** 1 Oncology, Guangxi University of Chinese Medicine, Nanning, CHN; 2 Oncology, Ruikang Hospital, Guangxi University of Chinese Medicine, Nanning, CHN

**Keywords:** adverse event, avelumab, cancer, faers, thrombocytopenia

## Abstract

Background

This study utilized data from the FDA Adverse Event Reporting System (FAERS) to investigate adverse drug events (ADEs) associated with avelumab, spanning from the third quarter of 2015 to the first quarter of 2024.

Methodology

We collected and normalized avelumab ADE data from Q3 2015 to Q1 2024. To ensure robust signal detection and accurate measurement of association strength, we employed key techniques, including the reporting odds ratio (ROR), proportional reporting ratio (PRR), Bayesian confidence propagation neural network (BCPNN), and empirical Bayes geometric mean (EBGM). These methods allowed for the comparison of event proportions, consideration of uncertainties, and adjustment for reporting variability, ensuring reliable signal quantification and analysis.

Results

In our review of 3,978 ADE reports, we classified unique preferred terms (PTs) into 22 systematic organ classifications (SOCs) to detail the range of avelumab-associated adverse reactions. The most commonly reported SOC was “General disease and administration site conditions,” with 868 cases reported (ROR = 1.22, PRR = 1.17, IC = 0.23, EBGM = 1.17). The second was nervous system disorders (294 cases) (ROR = 0.9, PRR = 0.91, IC = 0.14, EBGM = 0.91). The incidence of “Injury, poisoning and procedural complications” was 253 cases (ROR = 0.52, PRR = 0.55, IC = 0.86, EBGM = 0.55). This rank highlighted significant differences in the frequency and risk indicators of these diseases. It is worth noting that PTs with strong signals detected included thrombocytopenia (n = 35, ROR = 4.98, PRR = 4.95, IC = 2.31, EBGM = 4.95), hypothyroidism (n = 31, ROR = 15.49, PRR = 15.38, IC = 3.94, EBGM = 15.36), and renal impairment (n = 23, ROR = 3.93, PRR = 3.91, IC = 1.97, EBGM = 3.91).

Conclusions

While avelumab offers significant therapeutic benefits, its use carries potential adverse effects. Clinicians must remain vigilant in monitoring patients, particularly for severe symptoms, such as thrombocytopenia, hypothyroidism, and renal impairment, to ensure prompt intervention and minimize risks. Early detection of these and other potential events will help healthcare providers better manage and mitigate the risks associated with avelumab treatment.

## Introduction

Avelumab is a fully human monoclonal antibody that inhibits the interaction between programmed death-ligand 1 (PD-L1) on tumor cells and programmed cell death protein 1 (PD-1) on T cells, thus reducing immunosuppression in the tumor microenvironment and slowing tumor growth. It has been approved by the US FDA for the treatment of metastatic Merkel cell carcinoma and metastatic urothelial cancer (UC) that has progressed during or after platinum-based chemotherapy [1]. UC includes cancers originating from the urothelial lining of the bladder, renal pelvis, ureter, and urethra [2]. Bladder cancer alone accounts for 90% of UC cases and is the 10th most common cancer worldwide, with approximately 549,000 new cases and 200,000 deaths annually [3]. Around 5-15% of patients present with metastatic disease at diagnosis [4]. Extended follow-up from the JAVELIN Bladder 100 trial showed that avelumab offers long-term efficacy, with a median overall survival of 23.8 months from the start of maintenance treatment and 29.7 months from the initiation of first-line chemotherapy. Additional clinical datasets from non-interventional studies conducted in Europe, the United States, and Asia have further confirmed the efficacy of avelumab as first-line maintenance. Ongoing trials investigating avelumab-based combination regimens as maintenance treatments may further reshape the treatment landscape for advanced UC patients [5].

One study assessed the clinical efficacy of avelumab and its associated adverse events (AEs) in 3,935 cancer patients across 21 trials. The overall incidence of all-grade treatment-related AEs was 73.78%, with 14.44% reporting high-grade treatment-related AEs. Serious AEs occurred in 6.07% of patients, while fatal AEs were reported in 0.44%. The overall incidence of all-grade immune-related AEs was 17.86%, and high-grade immune-related AEs occurred in 3.22% of patients [6].

The FDA Adverse Event Reporting System (FAERS) is an important tool for collecting and analyzing adverse drug events (ADEs) related to drug use. In this study, data from FAERS was used to assess the safety and efficacy of avelumab, providing valuable insights into its overall risk profile. By employing a range of signal quantification techniques, the study goes beyond simple descriptive analysis to provide a comprehensive and detailed examination of the safety incidents associated with avelumab. These methods allow for a more precise identification of safety signals, which can then be further explored to determine potential risks associated with the drug. The use of multiple statistical and analytical methods ensured that the study results were robust and comprehensive, leading to a clearer understanding of how avelumab performs in real-world clinical settings. The application of these techniques in this study represents an important step in our knowledge of drug safety and highlights the potential of FAERS as a continuous pharmacovigilance tool.

## Materials and methods

Data source

This research utilized American Standard Code for Information Interchange report files from the FAERS database [7], spanning Q3 2015 to Q1 2024. Detailed information and data can be accessed through the website at https://fis.fda.gov/extensions/FPD-QDE-FAERS/FPD-QDE-FAERS.html, facilitating transparency and enabling reproducibility of our research findings. This period was specifically chosen because it coincides with the time following the drug’s market launch, ensuring the dataset reflects relevant post-market surveillance information. Once obtained, the data were carefully imported into R, a robust statistical computing environment, for thorough processing and analysis. R-enabled sophisticated data manipulation and statistical methods ensured that the analysis was both accurate and thorough.

Data extraction and analysis

To improve the demographics database and ensure its accuracy, duplicate reports were identified and removed. Only the most recent report for each unique case ID was kept, based on the report date, to ensure the analysis focused on the most relevant data. This method helped eliminate any redundant information from earlier submissions, maintaining a dataset that reflects the most up-to-date details. As a result, the analysis was more accurate, focusing on the latest available data and avoiding outdated or duplicate entries. Data linkage was performed using the “primaryid” field, with corrections made for discrepancies in age and weight information. Drug names were standardized using the Medex_UIMA_1.8.3 system to ensure consistency.

For additional analysis, reports that identified avelumab as the primary drug associated with ADEs were selected. These reports contained a wide range of detailed information, providing a comprehensive view of each case. Key data included the exact report date, enabling accurate chronological tracking of events. The patient’s age and gender were also recorded, offering valuable demographic insights for further analysis. The identity of the reporter was documented, ensuring accountability and allowing for follow-up if further clarification was needed. Furthermore, the geographical location of the report was noted, adding an important contextual layer that allowed for spatial analysis and the identification of potential regional patterns or trends. This detailed information enabled a deeper understanding of ADEs, supporting the exploration of demographic trends and geographic variations in the reported outcomes. It is essential for recognizing specific risk factors and gaining a broader understanding of the circumstances surrounding these AEs.

The study utilized four disproportionality methods to identify ADE signals, namely, reporting odds ratio (ROR) [8], proportional reporting ratio (PRR) [9], Bayesian confidence propagation neural network (BCPNN) [10], and empirical Bayes geometric mean (EBGM) [11]. Each of these methods has its own distinct strengths. ROR effectively minimizes bias from limited reports, providing more reliable results. PRR offers high specificity in detecting true signals. BCPNN excels in integrating and cross-validating data from multiple sources, enhancing robustness. MGPS is particularly suited to identifying signals from rare events that might be missed by other methods. By combining these methods, the study improves signal detection, validates results, and ensures reliable identification of safety signals. Cross-validation helps reduce false positives, while threshold adjustments and variance detection allow for the identification of rare adverse reactions. All methods use 2 × 2 contingency tables, as illustrated in Table 1, with relevant calculations and threshold values presented in Table 2.

**Table 1:** Four grid table. ADEs = adverse drug events

	Avelumab-related ADEs	Non-avelumab-related ADEs	Total
Avelumab	a	b	a + b
Non-avelumab	c	d	c + d
Total	a + c	b + d	N = a + b + c + d

**Table 2:** Four major algorithms used for signal detection. Equation a: the number of reports containing both the target drug and target adverse drug reaction; Equation b: the number of reports containing other adverse drug reactions of the target drug; Equation c: the number of reports containing the target adverse drug reaction of other drugs; Equation d: the number of reports containing other drugs and other adverse drug reactions. 95% CI = 95% confidence interval; N = the number of reports; χ^2^ = chi-squared; IC = information component; IC025 = the lower limit of 95% CI of the IC; E(IC) = the IC expectations; V(IC) = the variance of IC; EBGM = empirical Bayesian geometric mean; EBGM05 = the lower limit of 95% CI of EBGM; ROR = reporting odds ratio; PRR = proportional reporting ratio; BCPNN = Bayesian confidence propagation propensity for neural networks; MGPS = multi-item gamma Poisson shrinker

Algorithms	Equation	Criteria
ROR	ROR = ad/b/c	Lower limit of 95% Cl > 1, N ≥ 3
	95% CI = e^ln(ROR)±1.96(1/a+1/b+1/c+1/d)^^∧0.5^	
PRR	PRR = *a (c + d)/c / (a + b)*	PRR ≥ 2, χ^2^ ≥ 4, N ≥ 3
	*x^2^ = [(ad − bc) ^∧^2] (a + b + c + d)/ [(a + b) (c + d) (a + c) (b + d)]*	
BCPNN	*IC = *log*_2_ a (a + b + c + d) (a + c) (a + b)*	IC025 > 0
	*95%*CI* = *E(IC) *± 2 *V(IC)*^∧^0.5*	
MGPS	*EBGM = a (a + b + c + d)/ (a + c)/ (a + b)*	EBGM05 > 2
	*95%*CI* = e^ln (EBGM ) ±1.96(1/a+1/b+1/c+1/d)^^∧0.5^*	

Statistical analyses were conducted using Microsoft Excel 2022 (Microsoft Corp., Armonk, NY, USA), which served as the platform for calculating and interpreting key metrics related to AEs. In this context, higher values were used as important indicators of increased signal intensity. This increase in signal intensity was not random but represented a stronger association between the drug and the reported AEs. By interpreting these higher values as reflecting a stronger connection, the analysis could identify cases where the drug’s relationship with AEs was particularly significant. This approach made the findings clearer, allowing for easier identification of drugs that might be linked to more frequent or severe adverse outcomes. The interpretation of these values was crucial for assessing the drug’s overall risk profile in clinical settings. Using this method, it became possible to differentiate signals and quantify the strength of the association, thereby enabling more confident identification of potential safety concerns. Additionally, Microsoft Excel facilitated straightforward data visualization and organization, improving the clarity and interpretability of the results.

Filtering and categorization of signals

The initial screening phase of this study aimed to identify and select preferred terms (PTs) that were reported three or more times. This filtering criterion ensured that only terms with a significant number of reports were included for further analysis. To encode, categorize, and identify signals, the study utilized the Medical Dictionary for Regulatory Activities (MedDRA), with a specific focus on systematic organ classifications (SOCs) related to AE signals. MedDRA provided a standardized framework that ensured consistency and accuracy in classifying AEs, facilitating effective comparison and analysis across different datasets. This standardization was essential for maintaining uniformity in signal detection and for identifying specific SOCs most affected by the drug, ultimately contributing to a more precise understanding of the drug’s safety profile.

## Results

Key features of avelumab-related adverse event reports

**Figure 1:**
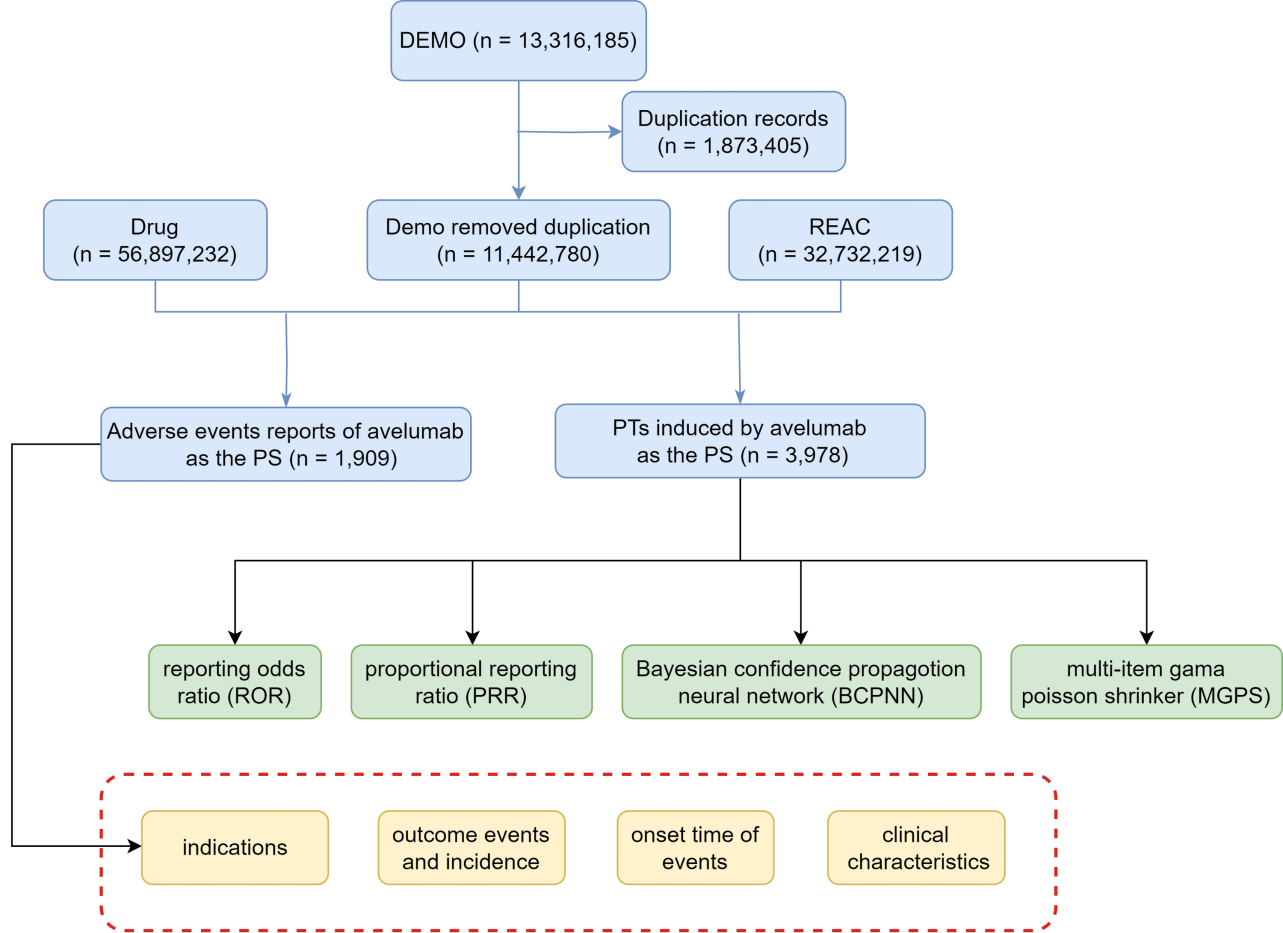
The flow diagram of selecting avelumab adverse events from FAERS database. PS = primary suspect drug; FAERS = FDA Adverse Event Reporting System; REAC = reaction file in FAERS, which lists adverse event reports. The number mentioned next to “REAC” (n = 32,732,219) indicates the total number of adverse event instances reported in the database during the specified period.

**Figure 2:**
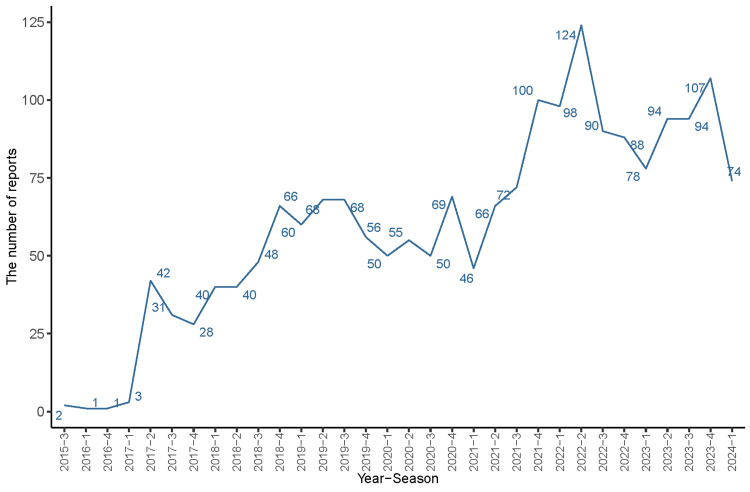
The number of adverse events reported quarterly after the marketing of avelumab.

**Figure 3:**
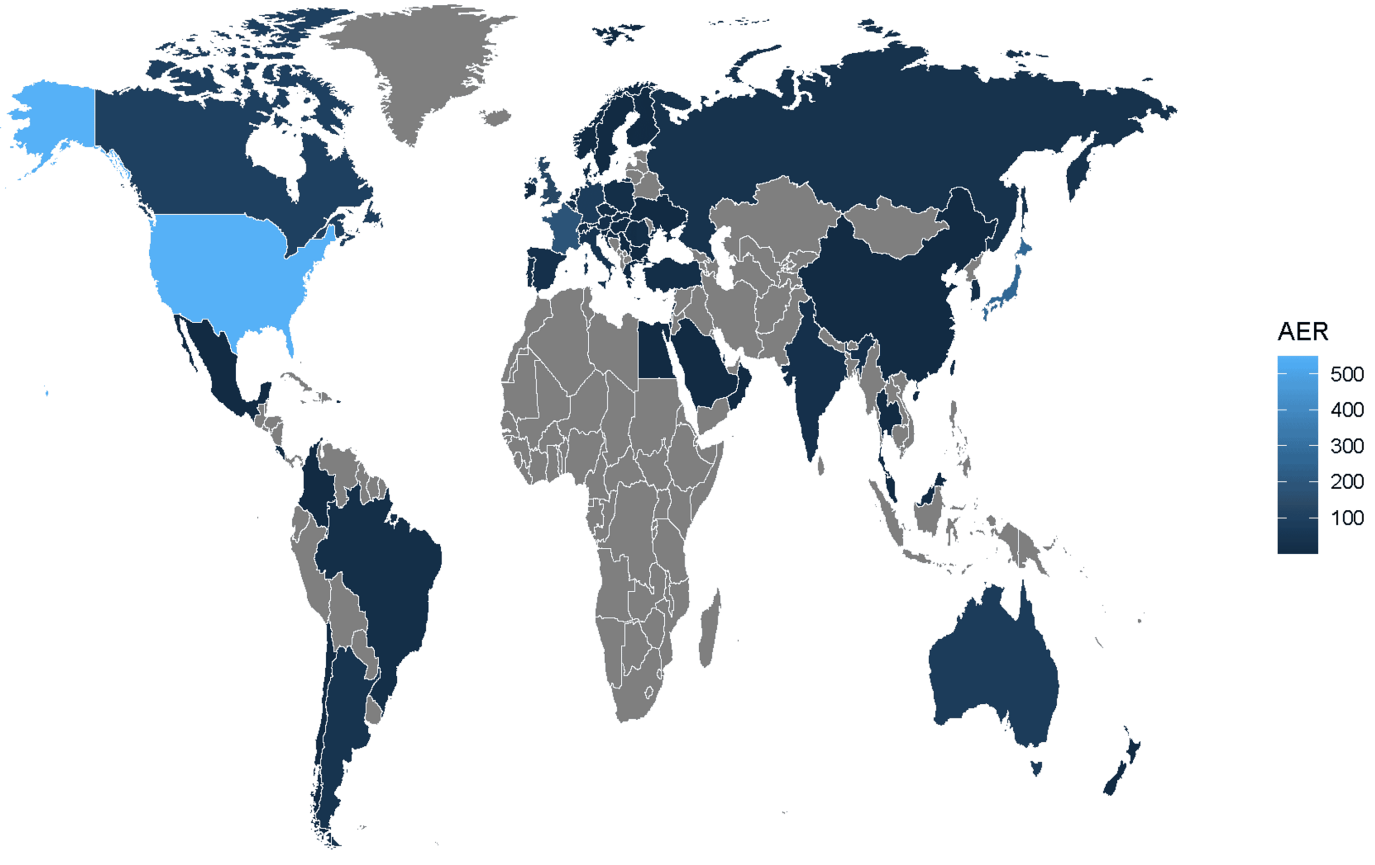
Global distribution of avelumab-related adverse reactions. AER = adverse event report

The study extracted 11,442,780 AE reports from the FAERS database, spanning from Q3 2015 to Q1 2024. Of these, avelumab was identified as the primary suspect in 3,978 ADE reports. Figure 1 illustrates the process for identifying avelumab-related AEs within the FAERS database, while Figure 2 highlights the quarterly collection and recording of these reports post-market, emphasizing the continuous monitoring of its safety profile. As shown in Figure 3, most reports of adverse reactions to avelumab came from the United States, followed by Japan and France.

A significant challenge in this study was the absence of age data in 23% of the reports, which hindered the ability to analyze age-related trends in AEs. This missing information limited our capacity to assess age-specific risks, introducing uncertainty into the findings. As a result, potential age-related risks could not be fully evaluated, underscoring the need for more complete data collection in future studies. However, in the reports that included age information, the most commonly reported age group was 65 to 75 years old.

The primary sources of AE reports were physicians (70.40%), pharmacists (13.93%), and consumers (10.06%), with the majority (28.81%) originating from the United States. Reports were more common among men (63.59%) than women (26.24%). Regarding the route of administration, intravenous administration (44.84%) was associated with the highest number of AEs, followed by other routes (40.39%). AEs were most frequently reported within two months or more of medication (29.85%), followed by within a week of medication (14.59%). However, the timing of AEs was unclear in 31.20% of cases, limiting a full assessment of risks related to the duration of medication. Regarding clinical outcomes, the highest incidence of AEs led to hospitalization (34.81%), followed by “other serious” outcomes (33.02%). Further details are provided in Table 3.

**Table 3:** Basic information on ADEs related to avelumab from the FAERS database. ADE = adverse drug event; FAERS = FDA Adverse Event Reporting System

Factors	Number of events (%)
Year	
2015	2 (0.10)
2016	2 (0.10)
2017	104 (5.45)
2018	194 (10.16)
2019	252 (13.20)
2020	224 (11.73)
2021	284 (14.88)
2022	400 (20.95)
2023	373 (19.54)
2024	74 (3.88)
Gender	
Female	501(26.24)
Male	1,214 (63.59)
Unknown	194 (10.16)
Age	
<18	2 (0.10)
18~45	41 (2.15)
45~65	434 (22.73)
65~75	528 (27.66)
>=75	465 (24.36)
Unknown	439 (23.00)
Reporter	
Physician	1,344 (70.40)
Pharmacist	266 (13.93)
Consumer	192 (10.06)
Other health professionals	96 (5.03)
Unknown	11 (0.58)
Reported countries	
Other	772 (40.44)
United States	550 (28.81)
Japan	273 (14.30)
France	181 (9.48)
United Kingdom	133 (6.97)
Route	
Intravenous	856 (44.84)
Other	771 (40.39)
Intravenous drip	282 (14.77)
Serious outcomes	
Hospitalization	700 (34.81)
Other serious	664 (33.02)
Death	505 (25.11)
life threatening	111(5.52)
Disability	27 ( 1.34)
Required intervention to prevent permanent impairment/damage	4 (0.20)
Adverse event occurrence time - medication date (days)	
<7	194 (14.59)
7~28	167 (12.56)
28~60	157 (11.80)
>=60	397 (29.85)
Unknown	415 (31.20)

Detection of signals based on systematic organ classification levels

The study conducted a comprehensive analysis of avelumab-related AE reports, identifying AE in 22 different SOCs, as shown in Table 4. This comprehensive assessment gave us a broad understanding of the scope and frequency of drug-related adverse reactions, highlighting which organ systems are most commonly affected. By categorizing the AEs of these different SOCs, the study aimed to provide a clearer and more detailed safety profile of avelumab to better understand its impact on patients. The most commonly affected SOCs were “General disease and administration site conditions,” with 868 cases reported (ROR = 1.22, PRR = 1.17, IC = 0.23, EBGM = 1.17). The second was nervous system disorders (294 cases) (ROR = 0.9, PRR = 0.91, IC = 0.14, EBGM = 0.91). “Injury, poisoning and procedural complications” comprised 253 cases (ROR = 0.52, PRR = 0.55, IC = 0.86, EBGM = 0.55).

**Table 4:** The signal strength of ADEs of avelumab at the SOC level in the FAERS database. ROR = reporting odds ratio; PRR = proportional reporting ratio; EBGM = empirical Bayes geometric mean; IC = information component; SOC = systematic organ classification; 95% CI = 95% confidence interval; ADE = adverse drug event; FAERS = FDA Adverse Event Reporting System

SOC	Case reports	ROR (95% CI)	PRR (95% CI)	Chi-square	IC (IC025)	EBGM (EBGM05)
General disorders and administration site conditions	868	1.22 (1.13, 1.32)	1.17 (1.1, 1.24)	27.62	0.23 (0.13)	1.17 (1.1)
Gastrointestinal disorders	324	0.95 (0.84, 1.06)	0.95 (0.86, 1.05)	0.94	-0.07 (-0.24)	0.95 (0.86)
Respiratory, thoracic, and mediastinal disorders	310	1.68 (1.49, 1.88)	1.62 (1.47, 1.79)	78.04	0.7 (0.53)	1.62 (1.47)
Nervous system disorders	294	0.9 (0.8, 1.01)	0.91 (0.81, 1.02)	3.14	-0.14 (-0.31)	0.91 (0.82)
Investigations	284	1.19 (1.05, 1.34)	1.17 (1.04, 1.32)	7.82	0.23 (0.06)	1.17 (1.06)
Injury, poisoning, and procedural complications	253	0.52 (0.46, 0.59)	0.55 (0.49, 0.62)	103.73	-0.86 (-1.04)	0.55 (0.5)
Musculoskeletal and connective tissue disorders	182	0.85 (0.73, 0.98)	0.85 (0.74, 0.97)	4.9	-0.23 (-0.44)	0.85 (0.75)
Infections and infestations	165	0.72 (0.62, 0.84)	0.73 (0.62, 0.85)	17.07	-0.45 (-0.67)	0.73 (0.64)
Skin and subcutaneous tissue disorders	158	0.65 (0.56, 0.76)	0.67 (0.57, 0.78)	28.15	-0.59 (-0.82)	0.67 (0.58)
Cardiac disorders	146	1.74 (1.47, 2.05)	1.71 (1.46, 2)	43.91	0.77 (0.54)	1.71 (1.49)
Renal and urinary disorders	128	1.58 (1.32, 1.88)	1.56 (1.31, 1.86)	26.27	0.64 (0.39)	1.56 (1.35)
Metabolism and nutrition disorders	122	1.47 (1.23, 1.76)	1.45 (1.22, 1.73)	17.69	0.54 (0.28)	1.45 (1.25)
Hepatobiliary disorders	114	3.44 (2.86, 4.15)	3.37 (2.83, 4.02)	191.64	1.75 (1.49)	3.37 (2.88)
Blood and lymphatic system disorders	114	1.69 (1.41, 2.04)	1.67 (1.4, 1.99)	31.51	0.74 (0.48)	1.67 (1.43)
Endocrine disorders	111	10.65 (8.82, 12.86)	10.38 (8.7, 12.38)	941.99	3.37 (3.1)	10.37 (8.85)
Vascular disorders	111	1.4 (1.16, 1.69)	1.39 (1.17, 1.66)	12.29	0.47 (0.2)	1.39 (1.19)
Immune system disorders	57	1.14 (0.88, 1.48)	1.14 (0.88, 1.47)	0.99	0.19 (-0.19)	1.14 (0.92)
Psychiatric disorders	49	0.21 (0.16, 0.28)	0.22 (0.17, 0.29)	146.29	-2.2 (-2.6)	0.22 (0.17)
Eye disorders	37	0.45 (0.32, 0.62)	0.45 (0.33, 0.62)	24.76	-1.14 (-1.6)	0.45 (0.35)
Ear and labyrinth disorders	9	0.49 (0.26, 0.95)	0.49 (0.26, 0.94)	4.69	-1.02 (-1.91)	0.49 (0.29)
Reproductive system and breast disorders	5	0.17 (0.07, 0.4)	0.17 (0.07, 0.41)	20.75	-2.57 (-3.73)	0.17 (0.08)
Congenital, familial, and genetic disorders	4	0.35 (0.13, 0.94)	0.35 (0.13, 0.93)	4.8	-1.51 (-2.77)	0.35 (0.15)

Signal detection based on preferred term levels

In this study, four algorithms were used at the PT level to analyze adverse drug reactions and assess their adherence to various screening criteria. Table 5 shows these PTs and classifies them according to the number of reports. This classification makes it easy to compare the frequency of different AEs, highlighting the most frequently reported PTs. Such classifications provide valuable insights into the relative incidence of specific drug AEs and contribute to a clearer picture of the overall safety of the drug. The results showed that some PTs showed strong signal strength, including thrombocytopenia (n = 35, ROR = 4.98, PRR = 4.95, IC = 2.31, EBGM = 4.95), hypothyroidism (n = 31, ROR = 15.49, PRR = 15.38, IC = 3.94, EBGM = 15.36), and renal impairment (n = 23, ROR = 3.93, PRR = 3.91, IC = 1.97, EBGM = 3.91).

**Table 5:** PT distribution of avelumab-related adverse reactions. ROR = reporting odds ratio; PRR = proportional reporting ratio; EBGM = empirical Bayes geometric mean; IC = information component; PT = preferred term; 95% CI = 95% confidence interval

PT	Case reports	ROR (95% CI)	PRR (95% CI)	Chi-square	IC (IC025)	EBGM (EBGM05)
Chills	65	8.83 (6.91, 11.29)	8.7 (6.88, 11.01)	443.58	3.12 (2.77)	8.7 (7.08)
Interstitial lung disease	63	20.1 (15.67, 25.79)	19.8 (15.35, 25.55)	1,122.58	4.3 (3.95)	19.75 (16.03)
Thrombocytopenia	35	4.98 (3.57, 6.95)	4.95 (3.55, 6.91)	110.45	2.31 (1.83)	4.95 (3.74)
Hypothyroidism	31	15.49 (10.88, 22.07)	15.38 (10.81, 21.89)	416.29	3.94 (3.44)	15.36 (11.42)
Myositis	24	48.16 (32.21, 72.03)	47.88 (32.35, 70.86)	1,095.38	5.57 (5)	47.61 (34)
Renal impairment	23	3.93 (2.61, 5.92)	3.91 (2.59, 5.9)	49.93	1.97 (1.39)	3.91 (2.78)
Neuropathy peripheral	22	3.32 (2.18, 5.04)	3.3 (2.19, 4.98)	35.39	1.72 (1.13)	3.3 (2.33)
Pneumonitis	21	11.07 (7.21, 17)	11.01 (7.15, 16.95)	191.05	3.46 (2.85)	11 (7.68)
Myocarditis	21	27.17 (17.68, 41.74)	27.03 (17.56, 41.6)	524.78	4.75 (4.15)	26.94 (18.81)
Colitis	20	7.8 (5.03, 12.11)	7.77 (5.05, 11.96)	117.94	2.96 (2.34)	7.76 (5.37)
Respiratory failure	19	4.43 (2.82, 6.95)	4.41 (2.81, 6.92)	50.14	2.14 (1.51)	4.41 (3.02)
Pulmonary embolism	19	3.93 (2.5, 6.16)	3.91 (2.49, 6.14)	41.21	1.97 (1.33)	3.91 (2.68)
Hepatic function abnormal	19	8.35 (5.32, 13.11)	8.32 (5.3, 13.06)	122.22	3.05 (2.42)	8.31 (5.7)
Oxygen saturation decreased	17	4.31 (2.68, 6.94)	4.29 (2.68, 6.87)	42.98	2.1 (1.43)	4.29 (2.88)
Pleural effusion	15	4.1 (2.47, 6.82)	4.09 (2.46, 6.81)	35.07	2.03 (1.32)	4.09 (2.68)
Hyperthyroidism	14	14.45 (8.54, 24.43)	14.4 (8.48, 24.45)	174.3	3.85 (3.11)	14.38 (9.26)
Hematuria	14	6.37 (3.77, 10.76)	6.35 (3.74, 10.78)	63.05	2.67 (1.93)	6.34 (4.09)
Alanine aminotransferase increased	13	4.04 (2.34, 6.96)	4.03 (2.33, 6.98)	29.59	2.01 (1.25)	4.03 (2.55)
Hypoxia	12	5.4 (3.06, 9.52)	5.39 (3.05, 9.52)	42.86	2.43 (1.64)	5.38 (3.35)
Blood creatine phosphokinase increased	12	8.66 (4.91, 15.27)	8.64 (4.89, 15.25)	81.02	3.11 (2.32)	8.63 (5.37)
Hepatitis	12	8.04 (4.56, 14.17)	8.02 (4.54, 14.16)	73.68	3 (2.22)	8.01 (4.99)
Adrenal insufficiency	12	14.31 (8.12, 25.23)	14.27 (8.08, 25.19)	147.87	3.83 (3.05)	14.25 (8.86)
Palmar-plantar erythrodysesthesia syndrome	12	7.25 (4.11, 12.78)	7.23 (4.1, 12.76)	64.42	2.85 (2.07)	7.23 (4.5)
Aspartate aminotransferase increased	11	4.17 (2.31, 7.55)	4.17 (2.32, 7.51)	26.47	2.06 (1.24)	4.16 (2.54)
Thyroiditis	11	50.27 (27.77, 91.02)	50.14 (27.85, 90.27)	526.57	5.64 (4.82)	49.84 (30.33)
Pancreatitis	11	4.06 (2.24, 7.33)	4.05 (2.25, 7.29)	25.26	2.02 (1.2)	4.05 (2.47)
Troponin increased	10	22.08 (11.86, 41.1)	22.03 (11.77, 41.25)	200.22	4.46 (3.6)	21.97 (13.06)
Hepatotoxicity	10	6.46 (3.47, 12.01)	6.44 (3.44, 12.06)	45.96	2.69 (1.83)	6.44 (3.83)
Thyroid disorder	10	9.77 (5.25, 18.19)	9.75 (5.21, 18.26)	78.47	3.28 (2.43)	9.74 (5.79)
Cytokine release syndrome	10	7.96 (4.28, 14.81)	7.94 (4.24, 14.87)	60.66	2.99 (2.13)	7.94 (4.72)

## Discussion

Monitoring the actual use of drugs and associated adverse reactions after marketing is essential to ensure safety and efficacy. This study analyzed the adverse effects of avelumab using FAERS data from the third quarter of 2015 to the first quarter of 2024. Through detailed analysis, this study not only validates previously known safety concerns but also identifies potential new risks associated with these drugs. These findings provide more detailed and accurate information to support medical practice and inform public health decisions.

In this study, a substantial proportion of the dataset was found to be missing crucial information, particularly concerning patient age and the timing of AEs following medication administration. This represents a significant limitation in the overall analysis. The absence of comprehensive age data hindered our ability to perform a detailed investigation into the incidence of AEs across different age groups, thereby restricting our understanding of how age may influence the occurrence of adverse effects. Additionally, the lack of precise information on the timing of post-medication AEs made it impossible to conduct a thorough assessment of the potential risks associated with the timing of the drug administration. This gap in the data further limits our ability to draw reliable conclusions about the relationship between the timing of medication and the onset of AEs. In light of these limitations, future studies should prioritize the collection of accurate and complete data regarding both patient age and the precise timing of AEs after medication use. This would provide a more comprehensive understanding of the safety profile of the drug across different patient populations and help to identify any age-related or time-dependent risks that may not have been evident in the current study.

In addition, it is important to highlight a notable difference in the occurrence of AEs between male and female patients receiving avelumab. This distinction suggests the possibility of gender-related factors influencing the frequency or nature of AEs. Interestingly, a significant proportion of the reports (28.81%) originated from the United States, which points to a potential regional or cultural pattern in the reporting of AEs. This concentration of reports in a specific geographic location raises questions about whether these trends are indicative of regional or cultural biases that could influence how AEs are identified, documented, and reported. To better understand the underlying causes of these disparities, further investigation is required. Specifically, it would be valuable to examine whether such patterns are reflective of actual differences in drug response between regions and genders, or if they stem from factors such as regional healthcare practices, differences in reporting mechanisms, or cultural variations in the recognition and reporting of AEs. Understanding these dynamics is crucial for ensuring a more accurate and equitable assessment of avelumab’s safety across diverse populations.

In the phase 3 JAVELIN Bladder 100 trial, avelumab first-line maintenance and best supportive care significantly prolonged overall survival and progression-free survival versus best supportive care alone in patients with advanced urothelial carcinoma who were progression-free following first-line, platinum-based chemotherapy, leading to regulatory approval in various countries [12]. Avelumab is a PD-L1 inhibitor, resulting in restoration of a cytotoxic, antitumor T-cell response [13]. Avelumab inhibits PD-L1/PD-1 interaction to disinhibit T-cells and removes the suppression of T-cell activity, whose response can be assessed by evaluating the release of interferon-gamma [14]. Avelumab does not modify the PD-L2/PD-1 pathway, permitting the continuity of PD-L2-arbitrated homeostasis [15]. Moreover, the interaction of PD-L1 with a second inhibitory receptor, B7.1, is inhibited by avelumab; this receptor might be expressed on T-cells and antigen-presenting cells [16]. By blocking the interaction between B7.1 and PD-1 on T-cells and with PD-L1 on antigen-presenting cells within the lymph nodes or tumor microenvironment, avelumab might reactivate T-cells and the production of cytokines [17]. Furthermore, because of its native IgG1 crystallizable fragment domain, avelumab conserves the ability to engage natural killer cells with the Fc-γ receptor to induce tumor-targeted, antibody-dependent, cell-mediated cytotoxicity in vitro [18]. This capacity to promote innate immune interactions against tumor cells makes avelumab exclusive among anti-PD-L1 or anti-PD1 antibodies in ongoing clinical trials.

AEs associated with avelumab have been described in existing literature; however, the underlying mechanisms responsible for these events remain largely speculative. While several hypotheses have been proposed, there is insufficient conclusive evidence to definitively identify the exact cause of these adverse effects. Therefore, further research is urgently needed to elucidate the mechanisms through which avelumab may trigger these AEs, which would significantly improve our understanding of the drug’s safety profile. Until such mechanisms are clearly established, clinicians need to remain vigilant in monitoring patients who are undergoing treatment with avelumab. This proactive approach should include closely tracking any emerging adverse reactions and intervening in a timely and effective manner to mitigate potential risks. Ensuring the safety of patients requires a careful balance between therapeutic efficacy and managing the adverse effects of the drug, and, as such, clinical vigilance and prompt response to adverse reactions will play a critical role in safeguarding patient health.

Although this study has provided valuable insights into the safety profile of avelumab, it is not without its limitations. One of the primary issues is the voluntary nature of reporting to the FAERS. As reporting is not mandatory, there is a possibility that the database may not fully capture all AEs associated with avelumab, potentially leading to an incomplete representation of the drug’s safety profile. This underreporting could skew the understanding of the drug’s overall risk. Additionally, the statistical methods employed in this study were designed to detect potential safety signals rather than to definitively confirm the actual risks associated with avelumab. This distinction is important because identifying safety signals does not necessarily equate to proving a causal relationship between the drug and the observed AEs. Another significant limitation is the frequent absence of comprehensive and detailed patient information in the FAERS database, which further complicates the interpretation of the data. The lack of critical demographic and clinical details, such as underlying conditions, concomitant medications, and patient histories, can hinder the ability to assess the full context of the reported AEs. Given these limitations, it is clear that further research is required to validate the findings presented in this study and to provide a more nuanced understanding of the AEs associated with avelumab. Future studies should aim to address these gaps in data and methodology to ensure a more robust evaluation of the drug’s safety. Nevertheless, despite these challenges, this study underscores the importance of continuous and vigilant monitoring of AEs in clinical practice, as such surveillance is crucial for identifying potential safety concerns in real-world settings and ensuring patient safety.

## Conclusions

In summary, our study used data from the FAERS database from the third quarter of 2015 to the first quarter of 2024 to provide a comprehensive analysis of avelumab-associated AEs. By applying various signal quantification techniques, we analyzed 3,978 ADE reports related to avelumab and identified PTs distributed across 22 SOCs. The most commonly reported SOCs are “General disorders and administration site conditions,” “Nervous system disorders,” and “Injury, poisoning and procedural complications.” These observations are consistent with the known therapeutic effects and side effects of avelumab. The agreement between the reported SOC and the expected effects suggests that these AEs may be related to the pharmacological action of the drug, providing valuable insights into common safety issues that may arise during treatment. Notably, strong adverse reaction signals such as thrombocytopenia, hypothyroidism, and renal impairment were observed. While avelumab offers therapeutic benefits, our findings highlight the need for clinicians to be vigilant about potential adverse effects. The study also highlights the value of post-marketing surveillance using databases such as FAERS to continuously evaluate drug safety profiles and guide clinical practice. Despite the limitations of the voluntary reporting system, such as incomplete data and reporting biases, our analysis provides important insights into avelumab’s safety profile. This highlights the need for further research to confirm these findings and deepen our understanding of ADEs. Ultimately, our research helps to improve the safe and effective use of avelumab in clinical practice, ensuring that its risks are controlled and its benefits maximized.
